# Limitations of Systemic Oncological Therapy in Breast Cancer Patients with Chronic Kidney Disease

**DOI:** 10.1155/2020/7267083

**Published:** 2020-05-18

**Authors:** Anna Bednarek, Joanna Mykała-Cieśla, Katarzyna Pogoda, Agnieszka Jagiełło-Gruszfeld, Michał Kunkiel, Mateusz Winder, Jerzy Chudek

**Affiliations:** ^1^Department of Internal Diseases and Oncological Chemotherapy, Faculty of Medical Sciences in Katowice, Medical University of Silesia, Katowice 40-027, Poland; ^2^Department of Breast Cancer and Reconstructive Surgery, Maria Sklodowska-Curie National Research Institute of Oncology, Warszawa 02-034, Poland

## Abstract

Breast cancer is the most common malignancy, affecting middle-age and older women frequently suffering from other chronic diseases, including chronic kidney disease. The risk of breast cancer development in women on renal replacement therapy (peritoneal dialysis and haemodialysis) is higher than in the general population. Chronic kidney disease does not limit surgical treatment or radiotherapy; however, it affects the pharmacokinetics of drugs used in the systematic treatment to a different extent, increasing their toxicity and the risk of adverse drug reactions. This article summarizes the current knowledge (published studies accessed through PUBMED) on drugs used in chemotherapy, hormone therapy, anti-HER2 drugs, CDK4/6 inhibitors, PARP inhibitors, and immune therapy in breast cancer patients undergoing dialysis. We discuss the data, the optimal choice of the chemotherapeutic protocol, and the administration of drugs in a specific time relation to the haemodialysis session to ensure the most effective and safe treatment to breast cancer patients.

## 1. Introduction

The incidence of chronic kidney disease (CKD) and its terminal stage—end-stage kidney disease (ESKD)—in the population increases due to the prolongation of human life. An increase in the number of patients with ESKD treated with renal replacement therapy is observed in the group of 60 years old and older. Haemodialysis (HD) is the primary method of renal replacement therapy that has been used since the early 1960s. The number of patients on dialysis differs substantially across countries and regions and is affected by access to health care [1]. The number of HD patients worldwide exceeds 1 million worldwide and is steadily growing. In Poland, this method is used for more than 20,000 patients yearly, including about 9,000 women [2]. The slowly improving survival of patients treated with renal replacement therapy increases the chance for cancer development, which results in a higher incidence of breast cancer in this population [3, 4].

The US Renal Data System indicates an increased risk of cancer, including breast cancer, among HD patients. In the years 1996–2009, 3552 HD women were diagnosed with breast cancer, that is, 42% more than in the general population [5]. In addition, a larger half (52.9%) of patients starting anticancer treatment had abnormal renal function (eGFR < 60 m/min/1.73 m^2^) and required anticancer drug dose adjustments. However, the lack of an appropriate drug dosage adjustment was related to a reduced overall survival [6].

Breast cancer is the most common malignancy in women worldwide, also in Poland (22% of malignancies in women). Globally, every eighteenth woman develops breast cancer over a lifetime [7, 8]. CKD is not listed among the most important risk factors for breast cancer, such as age, family history of breast cancer (*BRCA1* and *BRCA2* mutations), early menarche, late menopause, late pregnancy, long-term hormone replacement therapy (HRT), exposure to ionizing radiation, and some benign breast proliferative diseases [2, 9].

The management of breast cancer depends on the stage of cancer and includes surgery, which may be preceded or followed by chemotherapy or radiation therapy, or both. Oestrogen and progesterone receptor-positive cancers are often treated with hormone-blocking therapy over courses of several years. Monoclonal antibodies to HER2 (human epidermal growth factor receptor 2) receptors are used in patients with overexpression of these receptors on cancer cells [9, 10]. Advanced CKD (chronic kidney disease), HD, and peritoneal dialysis (PD) do not limit the possibility of surgical treatment and radiotherapy but have a significant impact on the pharmacokinetics of cytotoxic agents and other drugs used in oncological therapy due to the reduction/loss of renal clearance of drugs and their metabolites. The elimination of drugs during HD and PD depends on the diffusion that occurs through the semipermeable membrane and is limited by the protein binding potency.

Drugs with a low molecular mass (MM), up to 500 Da, and poorly binding to proteins are easily removed through a dialysis membrane and therefore should not be used immediately before the HD session (cyclophosphamide, 5-fluorouracil, and capecitabine). Modern HD techniques using synthetic highly permeable membranes (high-flux) and high ultrafiltration (haemodiafiltration) make it possible to remove significantly larger molecules with a MM greater than 1500 Da. However, the purification process is carried out only during 3 dialysis sessions per week.

This review aims to summarize the current knowledge on drugs used in adjuvant, neoadjuvant and palliative chemotherapy, hormone therapy, anti-HER2 drugs, CDK4/6 inhibitors, PARP (poly ADP-ribose polymerase) inhibitors, and immune therapy in breast cancer patients undergoing HD and PD. We discuss the data on how to optimally choose the chemotherapeutic protocol and administer the drugs in specific time relation to the HD procedure in this specific group of patients to ensure the most effective and safe treatment of breast cancer. The benefits of systemic therapy—potential improvement in progression-free survival and overall survival—should be weighed against an increased risk of toxicity as well as deterioration of the health-related quality of life.

This review was based on literature search in PUBMED (published before September 2019), concerning descriptions of the cases of HD patients with breast cancer, included data on the pharmacokinetics of chemotherapeutic agents in other than breast cancers malignancies, and comprised information given in the summaries of product characteristics, including the results of nonpublished studies.

## 2. Hormone Therapy

### 2.1. Tamoxifen

For over 20 years, tamoxifen (MM 371.5 Da) has been widely given in early and metastatic breast cancer patients with expression of oestrogen (ER) and/or progesterone (PgR) receptors [2].

Orally administered tamoxifen has an almost 100% bioavailability [11]. The molecule has high lipophilicity, and more than 95% of the drug is transported bound to proteins. Tamoxifen is metabolised by cytochrome P450 (CYP2D6 isoform) [12]. The main metabolites of tamoxifen are 4-hydroxy tamoxifen and N-desmethyltamoxifen. More than 60% of the drug dose is excreted unaltered in the faeces and only 9–14% with the urine. The plasma level of tamoxifen remains constant for 3-4 weeks when the daily supply is 20–40 mg once a day. The half-life of tamoxifen is 5–7 days, whereas of its metabolite N-desmethyltamoxifen—13 days. When the daily dose is 20 mg, serum concentrations of tamoxifen range from 164 to 494 ng/ml, and the mean concentration of N-desmethyltamoxifen is 226 ± 77 ng/ml [13]. The pharmacokinetics of tamoxifen depends on the age of the patient [14], but even in the case of older patients, it is not recommended to reduce the dose of tamoxifen.

Langenegger et al. [11] described the use of tamoxifen in an HD patient with breast cancer. This therapy was well tolerated by the patient. The plasma concentrations of the drug and its metabolite N-desmethyltamoxifen in dialysis patients were similar to those observed in non-HD patients. Therefore, tamoxifen pharmacokinetics does not force a modification of the drug dose in patients with CKD/ESKD, including the dialysed. Reducing the dose might limit the effectiveness of the treatment [15, 16]. Because of its high lipophilicity, the drug may be administered even shortly before the HD session.

### 2.2. Anastrozole

Aromatase inhibitors (anastrozole, letrozole, and exemestane) are used in early and metastatic breast cancer.

Anastrozole (MM: 293.4 Da) is a nonsteroidal aromatase inhibitor. There is an increased frequency of calcium and vitamin D deficiency in patients with CKD/ESKD and at the risk of renal osteodystrophy development. Therefore, bone mineral density (BMD) should be measured and vitamin D or its active metabolites therapy should be considered necessary before the initiation of the treatment with aromatase inhibitors [11].

Forty percent of the drug is bound to plasma proteins. As much as 85% of anastrozole is metabolised by the liver and excreted into the faeces, while only about 11% is excreted by the kidneys. Anastrozole half-life is long (41 h) [11]. According to Langenegger et al. serum anastrozole concentrations in HD patients are similar to those seen in patients with normal renal function, and the drug itself is well tolerated [11]. The results of this study indicate that in the case of CKD/ESKD patients, anastrozole may be used in the same dose as in patients with preserved kidney function. Due to the low MM and moderate affinity with plasma proteins, the drug should be taken after the HD session.

### 2.3. Letrozole

Letrozole (MM: 285.3 Da) is an aromatase inhibitor that is converted in the liver into an inactive metabolite carbinol (isoenzymes: 3A4 and 2A6 of cytochrome P450). There is no need to reduce the dose administered to older women [11].

Summary of drug characteristics informs that it is unnecessary to adjust the dosage for CKD patients whose creatinine clearance (CC) is greater than 30 ml/min. No dosage data are available for patients whose CC < 30 ml/min. There is a single report on treating an HD breast cancer patient with letrozole (with lapatinib) [17]. The therapy, without dose reduction, was tolerated well.

### 2.4. Exemestane

Exemestane (MM: 296.4 Da) strongly binds with proteins (90%) and is inactivated by the liver (metabolites are not biologically active). Only 1% of the administered dose is excreted unaltered into the urine. In patients whose CC < 30 ml/min. AUC (area under the concentration curve) was twice as large as in healthy volunteers. No dosage and safety data are available for patients whose eGFR <30 ml/min/1.73 m^2^ and those on renal replacement therapy. Taking into account the exemestane profile, no dose adjustment is required for CKD patients. However, due to the lack of data on pharmacokinetics and safety in dialysis patients, this drug should be used with caution, only when other therapeutic options are not available [18].

### 2.5. Fulvestrant

Fulvestrant is a selective oestrogen receptor degrader (SERD) with an MM of 606.9 Da highly (99%) bound to plasma lipoproteins, slowly metabolised via the same pathways as endogenous steroids in the liver and metabolites excreted into faeces. It is used in the metastatic setting. Its biological half-life is estimated at about 40 days. There is no published pharmacokinetic and safety data in dialysis patients concerning the use of fulvestrant. The negligible role of the kidney in the elimination of fulvestrant suggests its use in unchanged doses. This drug should be used with caution in severe CKD and ESKD patients [19].

### 2.6. Megestrol Acetate

Megestrol acetate (MM: 384.5 Da) is a synthetic progestin with the same physiologic effects as natural progesterone with antianorexic and anticachectic effect used in the therapy of progressive breast cancer for many years. The drug has a high affinity to albumins and therefore is not excreted by the kidneys. It is slowly metabolised by the liver, and its metabolites are excreted into faeces. The safety of this drug in dialysis patients with protein-wasting was shown in numerous trials [20]. There were few reported adverse drug reactions (suppressed cortisol levels, thrombophlebitis, and vaginal bleeding). Based on this experience, megestrol acetate can be recommended in the therapy of metastatic breast cancer.

In conclusion, taking into consideration the aforementioned data, giving adjuvant oestrogen deprivation therapy to breast cancer CKD/ESKD patients is considered quite safe. Due to limited safety data, patients with CKD/ESKD should receive tamoxifen or anastrozole or letrozole rather than exemestane. In patients with metastatic disease anastrozole, letrozole and megestrol acetate are considered safer than exemestane. Fulvestrant may be considered as the last-line of hormone therapy until the safety date is available in CKD/ESKD patients.

Except for anastrozole that should be administered after a dialysis session, all the drugs have high affinity to proteins and are not eliminated by dialysis. Therefore, thereis no need to specify any time relation to the dialysis session. In the case of fulvestrant, due to the intramuscular way of administration, the drug should be administered on nondialysis days.

### 2.7. Cyclin-Dependent Kinase 4/6 (CDK4/6) Inhibitors

CDK4/6 inhibitors added to hormone therapy significantly improve outcome in metastatic breast cancer patients. All three registered drugs (ribociclib, palbociclib, and abemaciclib) are small protein-bound molecules metabolised in the liver (CYP3A4) and excreted into faeces. The pharmacokinetic profiles do not justify a dose reduction of these drugs in CKD patients. However, the pharmacokinetic and safety data in “renal patients” are not available yet.

It was shown that CKD4/6 inhibitors may have some nephroprotective activity. The targeted inhibition of the CDK4/6 pathway was shown to ameliorate the kidney injury induced by cisplatin [21].

It should be mentioned that in about 40% of individuals, therapy with abemaciclib is associated with a reversible increase in serum creatinine concentration greater than 50% over the baseline level [22]. Abemaciclib was shown to inhibit renal tubular secretion of creatinine without changes in the measured glomerular filtration rate and the structural markers of kidney tubular injury (serum and urinary neutrophil gelatinase-associated lipocalin and urinary kidney injury molecule-1) [23].

## 3. Chemotherapy

Standard adjuvant and neoadjuvant chemotherapy consists of anthracycline-based multidrug regimens (doxorubicin, DOX or epirubicin, EPI with cyclophosphamide, CTX) and taxanes (docetaxel, DXL and paclitaxel, PXL), mainly in patients with a higher risk of recurrence. These drugs can be used also in advanced breast cancer. Other drugs for early or metastatic breast cancer are 5-fluorouracil (5-FU), capecitabine, methotrexate (MTX), vinorelbine (VRB), carboplatin, cisplatin (DDP), and gemcitabine [2, 10].

### 3.1. Anthracyclines

Doxorubicin (DOX) (MM: 543.5 Da) and epirubicin (EPI) (MM: 543.5 Da) are removed mostly by the liver and in a lesser degree excreted by the kidneys (15% and 10%, respectively). The AUC for DOX in HD patients is approximately 1.5 to 3 times higher than in patients with normal kidney function, and the HD removal of the drugs was low [24]. The impaired metabolism of DOX in patients with CKD is related to diminished activity of aldo-keto reductase in the kidney, an enzyme involved in the inactivation of the drug by C13 carbonyl reduction of doxorubicin to its inactive hydroxy metabolite doxorubicinol [25, 26].

For this reason, a 20% reduction of the DOX dose in patients with CKD seems reasonable. A dose reduction in dialysis patients is not recommended [27]. There are no data on EPI pharmacokinetics in HD patients. In the case of patients with CC < 30 ml/min, a reduced dose should be considered; however, the number of studies on the effectiveness of a reduced dose is very limited [28]. Gori et al. [29] presented a case report of a 51-year-old HD patient with breast cancer. In the case described, EPI therapy was well tolerated. No leukopenia, thrombocytopenia, and cardiotoxicity were observed (ejection fraction—LVEF—was stable).

CKD appears to worsen the cardiotoxicity of EPI. Russo et al. described a 12-month follow-up indicating an increased risk of total anthracyclines, taxanes, and trastuzumab cardiotoxicity in patients with an estimated glomerular filtration rate (eGFR) <60 ml/min/1.73 m^2^. In this group of patients, cardiac events were 52% more common than in those with eGFR ≥60 ml/min/1.73 m^2^ (38% vs. 25%) [30].

Because of the MM and no data on HD removal, DOX and EPI administration is recommended after the HD session or on nondialysis days.

### 3.2. Cyclophosphamide

Cyclophosphamide (CTX) (MM: 261.1 Da) is excreted in 50% to 70% by the kidneys within 48 hours, 32% of which is excreted in an unaltered form [24]. Nephrotoxicity is a very rare side effect (CKD is a risk factor). When excreted in the urine, CTX metabolites damage the epithelium of the urinary tract—especially in the bladder. Therefore, haemorrhagic cystitis is the most common dose-dependent adverse drug reaction to CTX [31].

The number of studies concerning CTX treatment in dialysis patients is very limited. In the available literature, a case report of a 48-year-old HD woman with breast cancer was described. In the case, the maximum plasma concentration of CTX was 49 *μ*g/ml, and the *in vivo* half-life was 67 h [24].

CTX is removed during HD. It should, therefore, be administered after HD or on nondialysis days. A 25% dose reduction is recommended [27].

### 3.3. Two-Drug Regimens (AC/EC)

AC regimen with a 20–25% reduction of the DOX dose can be considered safe in patients with CKD/ESKD, including HD patients [27]. Attention should be paid to the increased risk of cardiotoxicity. Due to the lack of EPI pharmacokinetic data, the EC regimen is not recommended for patients with ESKD and on HD. There are no data available to evaluate/compare the efficacy of these treatments in ESKD patients.

When using two-drug regimens in HD patients, chemotherapy should be administered on nondialysis days.

### 3.4. Paclitaxel and Docetaxel

Paclitaxel (MM: 853.9 Da) and its semisynthetic analogue—docetaxel—(MM: 807.9 Da) are microtubule antagonistic drugs. Both drugs strongly bind to proteins (>90%), primarily with albumin and alpha-1-glycoprotein [32]. They are metabolised in the liver by the cytochrome P450 and excreted into the bile. In a small amount, they are excreted into the urine [28]. The pharmacokinetics of a 135 mg/m^2^ dose of PXL in a 3-hour intravenous infusion in an HD patient was similar to that in patients with normal renal function [33]. The description of DXL pharmacokinetics is based on a single HD patient [34], indicating no effect of an impaired renal function on the elimination process. These data indicate the possibility of treating dialysis patients with the unaltered dosage of these drugs. Currently, in breast cancer patients, PXL is usually administered in a dose of 80 mg/m^2^ every week. The pharmacokinetics data for this dose are not available. However, as the pharmacokinetics of a larger dose of PXL is not affected by the kidney function, the alterations are not expected.

PXL was well tolerated by HD patients. Watanabe et al. described safe and effective use of PXL in a 40-year-old HD woman [33]. Good tolerance of DXL was also demonstrated in a 72-year-old HD patient with prostate cancer [34]. The authors proposed the use of DXL at an initial dose of 65 mg/m^2^, and its possible increase when the drug tolerance was good.

### 3.5. Anthracycline- and Taxane-Based Regimens

The results of the studies suggest that the AC ⟶ T (doxorubicin and cyclophosphamide followed by docetaxel) regimen can be used in CKD/ESKD (including HD) patients with breast cancer, with the already mentioned 20–25% dose reduction of DOX in HD patients. There are a lack of data enabling evaluation/comparison of the efficacy of these treatments in individuals on renal replacement therapy with nonrenal patients.

### 3.6. 5-Fluorouracil (5-FU)

5-FU (MM: 130 Da) is a pyrimidine antimetabolite. After intravenous administration, the half-life of 5-FU is about 16 minutes and depends on the dose of the drug. Only about 15% of the 5-FU dose is excreted unaltered into the urine [28].

It is believed that typical doses of 5-FU may be given to ESKD patients after the HD session or on nondialysis days.

### 3.7. Capecitabine

Capecitabine (MM: 359.3 Da) is a prodrug converted to 5-FU. Capecitabine and its active metabolites are excreted primarily by the kidneys, i.e., 96% of the administered dose is detected in the urine. The literature on the treatment of HD patients with capecitabine is limited. Jhaveri et al. described 12 patients with severe CKD or ESKD (CC <30 ml/min), among them two cases of HD patients were treated with capecitabine. Its toxicity was acceptable (low). Patients with ESKD were treated with a reduced dose (on average, up to 55% of the standard dose). Despite the dose reduction, a satisfactory response to the treatment was observed. The pharmacokinetics of the drug was evaluated in this study. The authors suggested the reduction of the drug dose by half [35]. The low number of observations in addition to the dose reduction suggests the need for strict monitoring of the drug toxicity (myelosuppression, hand-foot syndrome, and diarrhoea) after therapy initiation. The low MM of the drug and the lack of protein binding preclude capecitabine administration before the HD session.

### 3.8. Gemcitabine

Gemcitabine (MM: 263.2 Da) is quickly metabolised in the liver and the kidneys, with its renal filtration not exceeding 10%, as only 10% is bound to plasma proteins. Kidneys, as well as HD, remove the main noncytotoxic metabolite of gemcitabine—difluorodeoxyuridine (dFdU).

No significant toxicity and pharmacokinetic alterations of gemcitabine and dFdU were reported in CKD and ESKD patients receiving doses of up to 1200 mg/m^2^ compared to patients with a normal kidney function. A dose reduction of gemcitabine in CKD/ESKD patients is not required. The HD session should start not earlier than 6–12 hours after the infusion of chemotherapy [36]. There are no data concerning toxicity and efficacy in breast cancer patients with ESKD.

### 3.9. Carboplatin

Carboplatin (MM: 371.3 Da) is a second-generation of less nephrotoxic platinum analogues. Administered intravenously, carboplatin is not avidly protein bound initially, but the majority of the drug becomes protein bound within 24 h, and 55–70% of the drug is excreted by the kidney in the first 24 h [37]. According to the summary of product characteristics, carboplatin may be used in patients with CC > 20 ml/min, but the dose has to be reduced when CC is below 60 ml/min to prevent excessive myelotoxicity [38]. In so-called “renal patients,” the dose should be calculated according to the Calvert formula for the fixed AUC target [39]. However, not recommended by the manufacturers, carboplatin in a reduced dose may be used in dialysis patients. Few reports describe nonbreast cancer patients treated with carboplatin with a dose calculated according to the Calvert formula with CC taken as zero, corrected for the time interval between the infusion and the planed HD or PD session [40, 41]. Recently, AIOM guidelines suggest calculation of a carboplatin dose AUC × 25 mg in HD patients [27].

Both the HD and peritoneal procedures should be performed within 12–18 h after the carboplatin infusion before carboplatin has become bound to proteins and not dialyzable. Hiraike et al. demonstrated that the standard dose of carboplatin-targeted AUC was determined based on the Calvert formula, without any correction, and maintained quite normal pharmacokinetics, if the HD began an hour after the dose administration [42]. In the PD patient (CAPD), 20% of the dose is cleared via the dialysate, while the half-lives of carboplatin are double compared to patients with normal renal function [43].

Optimally, the carboplatin-based chemotherapy should be administered on dialysis days, shortly before the session to eliminate the cell breakdown products. However, for organisational reasons, the current guidelines recommend the administration of carboplatin on nondialysis days [27].

### 3.10. Cisplatin (CDDP)

CDDP (MM: 300 Da) is less frequently used than carboplatin platinoid in the palliative therapy of triple-negative breast cancer resistant to taxanes and anthracyclines [44]. The drug strongly binds to protein and is slowly eliminated from the circulation (T1/2 = 58.5–73 h) [45] mainly by the kidney and excreted with urine as an unchanged drug (15–75%) [44, 45]. Nephrotoxicity precludes its use in CKD patients; however, it may be used in dialysis ESKD patients [46]. The feasibility of cisplatin-based regimens was confirmed in nonbreast cancer patients [47, 48]. These data clearly show that CDDP doses 50–80 mg/m^2^ are quite well tolerated when starting the HD session within 30 min postinfusion. However, there are no data concerning CDDP safety and efficacy in dialysed breast cancer patients [49, 50].

### 3.11. Methotrexate (MTX)

Methotrexate (MM: 454.4 Da), which is a folic acid derivative, belongs to the group of antimetabolites. MTX is eliminated primarily by the kidneys. The excretion depends mainly on the dose and route of the drug administration. After intravenous administration, about 90% of the dose is eliminated unaltered from the body within 24 hours. No more than 10% of the dose is excreted into the bile. The MTX metabolite of the highest importance is 7-hydroxymethotrexate produced in the liver with aldehyde oxidase. The MTX half-life in the terminal elimination phase is between 3 and 10 hours in patients treated with low doses of MTX (less than 30 mg/m^2^), whereas in the case of patients receiving high doses of MTX, the terminal elimination half-life is 8 to 15 hours. Competition between MTX and other drugs excreted with the same mechanism may increase serum levels of this drug. Nonsteroidal anti-inflammatory drugs may interfere with MTX renal clearance and lead to toxic symptoms of the therapy. The common adverse drug reactions (ADRs) of high doses of MTX used in haematology (up to 6 g/day) include haematuria and acute kidney injury. During MTX treatment, its metabolites may precipitate in the kidney tubules. Therefore, intensive fluid therapy and alkalization of the urine to pH 6.5–7.0 are recommended during the treatment, e.g., with sodium bicarbonate (5 tablets × 625 mg every three hours) or acetazolamide (not recommended in CKD). High doses of MTX, i.e., above 1000 mg/m^2^, may cause acute renal failure, which worsens the elimination of the drug from the body. The incidence of ADRs increases with the dose [51].

The doses used in the therapy of breast cancer (CMF regimen) are much lower. However, Langleben et al. described the effects of severe toxicity following the first administration to a patient with breast cancer [52]. The reduction of toxicity can be achieved using daily high-flux dialysis [53].

The use of MTX in ESKD patients is not recommended if there is any other treatment option. In HD patients, the dose has to be reduced by 75% [27].

### 3.12. CMF Regimen (CTX, MTX, and FU)

This chemotherapy regimen is not recommended due to the poor tolerance of MTX by CRF and HD patients.

### 3.13. Vinorelbine (VRB)

Vinorelbine (778.9 Da) is removed from the body mainly by the liver. Only 8% of the administered dose is excreted unaltered by the kidneys [54]. There are no data on pharmacokinetics in CKD/ESKD and HD patients. There is a single report of VRB administration in a HD patient in a weekly dose of 25 mg/m^2^, resulting in severe leukopenia with pneumonia. The authors reduced the dose by 50% and that was well tolerated. However, it is uncertain whether the efficacy of the therapy was maintained. According to AIOM, a reduction of 25–33% should be considered [27].

In patients with metastatic disease and severe CKD or ESKD, the VRB + DOX, a rarely used regimen, should be debated in breast cancer treatment only when other therapies are no longer available.

## 4. Anti-HER2 Therapy: Tyrosine Kinase Inhibitors

### 4.1. Lapatinib

Lapatinib (MM: 851 Da) is a dual tyrosine kinase inhibitor that interrupts the HER2 and EGFR pathways registered for therapy of metastatic disease in combination with capecitabine. It highly bounds to albumin and undergoes extensive metabolism, primarily by CYP3A4 and CYP3A5 to a variety of oxidized metabolites excreted with the faeces. As lapatinib is not eliminated by the kidneys and is highly bound to plasma albumin, HD is not expected to enhance the elimination of the drug. A single case report suggested good tolerance of lapatinib with letrozole in an HD female with metastatic disease [17].

### 4.2. Neratinib

Neratinib (MM: 557 Da) is a tyrosine kinase inhibitor that together with their active metabolites irreversibly binds to EGFR, HER2, and HER4 reducing autophosphorylation of the receptors, and, as a consequence, blocks signal transduction. It is used in the extended adjuvant therapy for early-stage breast cancer with HER2 overexpression, after a 1-year therapy with trastuzumab [55]. The drug strongly binds to proteins (99%) and is eliminated by the liver and excreted (metabolites) mostly with the faeces (97%). Episodes of acute renal failure were reported as a consequence of diarrhoea and vomiting with dehydration [55].

The pharmacokinetics of the drug was not studied in dialysis patients, yet, but available data do not indicate the need for dose adjustment in patients with renal dysfunction. The safety data in kidney patients are not accessible.

## 5. Anti-HER2 Therapy: Monoclonal Antibodies and Conjugates

Passive immunotherapeutic strategies in the management of breast cancer overexpressing HER2 include the use of trastuzumab (recombinant monoclonal humanized IgG1 HER2 blocker antibody) and, more recently, pertuzumab (recombinant monoclonal humanized IgG1 antibody that blocks HER2 receptor dimerization) and T-DM1 (trastuzumab emtansine conjugate).

### 5.1. Trastuzumab

Trastuzumab treatment is generally well tolerated; however, it is associated with an increased risk of cardiac dysfunction. Nevertheless, its long-term side effects have not been fully assessed. Because of their cardiotoxicity, anthracyclines and trastuzumab should not be used simultaneously. Due to the nonlinear elimination of the drug (catabolism), the total clearance increases along with the decrease of its concentration. Therefore, the half-life of trastuzumab cannot be easily determined. T1/2 falls together with a decrease in the concentration between successive doses [56].

Available data do not indicate the need for a dose adjustment in patients with renal dysfunction [57]. It should be stressed that pharmacokinetic studies of this drug did not include patients with ESKD. Only two observations on treating HD patients with trastuzumab are available in the literature [57, 58]. In both cases, a clinical response was obtained with good drug tolerance. The authors pointed out that the therapeutic concentration of trastuzumab was reached, but no data were collected on the level of trastuzumab in the blood. Another case report is available on a 64-year-old HD patient treated with trastuzumab at a dose of 4 mg/kg—a loading dose—followed by 2 mg/kg every 7 days for one year. The data on the pharmacokinetics of trastuzumab were collected during the first course of the treatment. The maximum plasma concentration was 190 mg/l at a dose of 2 mg/kg, and the maximum plasma concentration ranged from 75 to 163 mg/l. The plasma concentration of trastuzumab did not decrease during HD [58]. It was constant and reached more than 20 mg/l, and it was within the therapeutic range recommended for patients treated for breast cancer. The collected data show that trastuzumab is not eliminated from the blood during HD due to its high MM (145 kDa). The pharmacokinetics of the drug, in this case, was similar to that observed in patients with a normal renal function.

In conclusion, the pharmacokinetics of trastuzumab in the treatment of breast cancer in HD patients does not undergo significant changes, but it may, however, imply an increased risk of cardiotoxicity, which should be verified in further studies. For this reason, the first control echocardiography should be performed earlier, 6–9 weeks after therapy initiation.

### 5.2. Trastuzumab Emtansine (T-DM1)

T-DM1 is an antibody-drug conjugate consisting of the monoclonal antibody—trastuzumab—covalently linked to a microtubule inhibitor—emtansine (DM1)—indicated for the treatment of patients with HER2-positive, unresectable, locally advanced, or metastatic breast cancer who had previously received trastuzumab and a taxane, separately or in combination. It is recently recommended also in patients with residual disease (non-pCR) after neoadjuvant therapy. T-DM1 catabolites (DM1, Lys-MCC-DM1, and MCC-DM1) are mainly excreted by the liver (in bile) with a minimal elimination with urine due to high MM (148 kDa) [59]. The effect of GFR on T-DM1 clearance was not clinically relevant [60]. The pharmacokinetics of the drug was not studied in dialysis patients, yet. The safety data in kidney patients are not available. However, increased cardiotoxicity can be expected, similarly to its component–trastuzumab.

According to the summary of product characteristics, it is not necessary to modify the dose of T-DM1 in patients with mild-to-moderate renal impairment [59].

### 5.3. Pertuzumab

This drug is used in combination with trastuzumab and DXL in patients with metastatic HER2-positive breast cancer or unresectable local recurrence. It is also used in neo/adjuvant therapy in early breast cancer. Pertuzumab is a humanized monoclonal antibody with a high MM that prevents it from being removed through the HD membrane. Proteolytic degradation constitutes the main mechanism of the elimination of pertuzumab, as other antibodies, from the circulation.

According to the summary of product characteristics, it is not necessary to modify the dose of pertuzumab in patients with mild or moderate renal impairment. Because of the limited number of pharmacokinetic data, there is no recommendation for administering the drug to patients with severe renal impairment including ESKD. Regardless of the coexistence of CKD, during the administration of pertuzumab, the left ventricular ejection fraction should be monitored due to the risk of congestive heart failure, which is an infrequent complication of this therapy [61].

## 6. Poly ADP-Ribose Polymerase (PARP) Inhibitors

Both olaparib (MM: 435 Da) and talazoparib (MM: 380 Da) are PARP inhibitors—an enzyme involved in DNA repair. They are both approved for the therapy of germline BRCA-mutated, HER2-negative metastatic breast cancer [62, 63]. These small molecules are strongly bound to proteins. Olaparib is eliminated, mostly as metabolites, with the faeces and the urine in similar amounts. Due to the increased AUC (by 44%) and Cmax (by 26%) in patients with moderate renal impairment (CC 30–50 ml/min), a dose adjustment (25% decrease of the daily dose) is recommended [64]. Talazoparib is eliminated in an unchanged form by the kidneys [65]. Like in the case of olaparib, a 25% daily dose reduction of talazoparib is recommended in patients with moderate CKD (CC 30–60 ml/min) [66, 67]. For both PARP inhibitors, there are no sufficient pharmacokinetic data in patients with severe CKD and ESKD. High rates of myelotoxicity, including severe anaemia, may be expected in CKD patients.

## 7. Immune Therapy

Atezolizumab is a fully humanized, IgG1 isotype monoclonal antibody against the programmed cell death-ligand 1 (PD-L1). It was recently approved by the EMA and the FDA for treatment of advanced triple-negative breast cancer (plus nab-paclitaxel) [68]. The antibodies are slowly cleared from the circulation mainly by catabolism. There are no data on the safety of atezolizumab in CKD breast cancer patients. The only data came from the IMvigor210 study which involved 83 patients with locally advanced or metastatic urothelial carcinoma and decreased CC (30–60 mL/min) treated with atezolizumab without a dose reduction. The therapy was well tolerated [69]. Similarly, good tolerance of therapy was reported in a single HDpatient with metastatic urothelial cell carcinoma [70]. These data show no need for a dose adjustment in patients with mild-to-moderate CKD, and probably ESKD.

## 8. Summary

It should be noted that for the older drugs, the safety in HD patients is ensured in most cases (Table 1). There are insufficient data on treating HD patients with letrozole, paclitaxel, cyclophosphamide, and all newer medical products: CDK4/6 inhibitors, neratinib, PARP inhibitors, T-DM1, pertuzumab, and atezolizumab. A dose reduction is advisable for the administration of DOX, EPI, CTX, carboplatin, capecitabine, VNR, olaparib, and talazoparib. Presumably, also some other drug doses should be reduced due to an increased risk of myelotoxicity (e.g., CDDP) and cardiotoxicity (DXL). The only drug that should be avoided in HD patients is MTX.

It is important to emphasize the enhanced efficiency of the metabolic liver function in patients with ESKD [71] concerning certain drugs. However, it only slightly compensates the lost of excretion of chemotherapeutic agents into the urine.

Mainly for organisational reasons, but also to avoid sudden elimination of some drugs during the HD session, it is advisable to administer chemotherapy on nondialysis days.

## Figures and Tables

**Table 1:** Summary of pharmacokinetics and safety of systemic therapeutics for breast cancer in haemodialysis patients.

Drug	Molecular mass (Da)	Elimination	Dose reduction in haemodialysis patients	Safety	Administration in relation to haemodialysis session	Literature
Tamoxifen	371.5	60% with faeces, 9–14% with urine	Not indicated	Safe	Before HD	[11–16]
Anastrozole	293.4	85% with faeces, 11% with urine	Not indicated	Safe	After HD	[11]
Letrozole	285.3	90% with urine (metabolites)	Not specified	No data	NA	[17]
Exemestane	296.4	1% with urine	Not indicated	Safe	NA	[18]
Fulvestrant	606.9	<1% with urine, 90% with faeces (metabolites)	Not indicated	No data	Nondialysis days	[19]
Megestrol acetate	384.5	8% with urine, 90% with faeces (metabolites)	Not indicated	Safe	NA	[20]
Ribociclib	434.5	69% with faeces, 23% with urine	Not specified	No data	NA	[21]
Palbociclib	447.5	74% with faeces, 18% with urine	Not specified	No data	NA	[21]
Abemaciclib	506.6	81% with faeces, 3% with urine	Not specified	No data	NA	[21–23]
Docetaxel	807.9	75% with faeces, 6% with urine	Not specified	Safe	Before or after HD	[24,32,34]
Paclitaxel	853.9	1.3–12.6% with urine	Not specified	Safe	Before or after HD	[33,43,50]
Doxorubicin	543.5	15% with urine	Recommended dose reduction by 20%	Increased risk of cardiotoxicity	After HD	[25,26]
Epirubicin	543.5	10% with urine	Recommended dose reduction (creatinine > 450 µmol/l)	Increased risk of cardiotoxicity	After HD	[29]
Carboplatin	371.3	Almost all with urine	Recommended dose reduction (CC < 60 ml/min)	Increased risk of myelotoxicity	Nondialysis days	[37–43]
Cisplatin	300	Almost all with urine	Nonspecified in dialysis patients	Increased risk of nephrotoxicity in CKD patients. Increased risk of myelotoxicity in dialysis patients	After HD	[44–50,71]
Cyclophosphamide	261.1	50–70% with urine	Recommended dose reduction by 20%	Haemorrhagic cystitis (CKD independent)	After HD	[24,27,31]
5-Fluorouracil	130.1	15% with urine	Not indicated	Safe	After HD	[27,28]
Capecitabine	359.3	96% with urine	Recommended dose reduction by 50%	Safe (limited data)	Before HD	[35]
Gemcitabine	263.2	Liver, kidneys <10%	Not recommended	Safe	6–12 hours before HD	[27,36]
Methotrexate	454.4	90% with urine	No data	Increased myelotoxicity in dialysis patients, high-dose nephrotoxicity in CKD	After HD	[27,52,53]
Vinorelbine	778.9	8% with urine	Probably necessary (up to 50%)	Increased myelotoxicity (limited data)	After HD	[54]
Lapatinib	581.1	2% with urine, >90% with faeces (metabolites)	Not indicated	Safe (limited data)	NA	[17]
Neratinib	557	2% with urine, 97% with faeces (metabolites)	Not indicated	No data	NA	[55]
Olaparib	435.1	44% with urine, 42% with faeces (metabolites)	Recommended 25% dose reduction (CC < 50 ml/min)	No data	NA	[62,64]
Talazoparib	380.3	50% with urine, 14% with faeces	Recommended 25% dose reduction (CC < 60 ml/min)	No data	NA	[63,65–67]
Trastuzumab	145 kDa	No data	Not indicated	Increased risk of cardiotoxicity	NA	[56–58],
Trastuzumab emtansine	148 k	Mainly with faeces (metabolites)	Not indicated	Increased risk of cardiotoxicity	NA	[59]
Pertuzumab	148 k	No data	Not indicated	No data	NA	[61]
Atezolizumab	144 k	No data	Not indicated	Safe (very limited data)	NA	[68–70]

Abbreviations: CC, creatinine clearance; HD, haemodialysis; NA, not applicable.

## Additional Points

*Highlights.* (1) Growing population of dialysed women has increased the incidence of breast cancer. (2) Kidney disease affects the pharmacokinetics of drugs used in breast cancer treatment. (3) Pharmacokinetic changes in different drugs vary. (4) Increased toxicity of some drugs requires reduction of doses in dialysis patients. (5) Removal of drugs has to be included in the chemotherapy plan and dialysis schedule.
